# ICF-based multidisciplinary rehabilitation program for complex regional pain syndrome of the hand: efficacy, long-term outcomes, and impact of therapy duration

**DOI:** 10.1186/s12893-020-00982-7

**Published:** 2020-12-01

**Authors:** D. Kotsougiani-Fischer, J. S. Choi, J. S. Oh-Fischer, Y. F. Diehm, V. F. Haug, L. Harhaus, E. Gazyakan, C. Hirche, U. Kneser, S. Fischer

**Affiliations:** BG Trauma Center Ludwigshafen, Department for Hand-, Plastic- and Reconstructive Surgery, University of Heidelberg, Ludwig-Guttmann-Str. 13, 67071 Ludwigshafen, Germany

## Abstract

**Background:**

Complex regional pain syndrome (CRPS) is a rare but feared complication in hand surgery. Although multimodal therapy concepts are recommended, there is only low evidence on efficacy of such approaches. Furthermore, recommendations regarding therapy duration are lacking. Aim of this study was to validate the efficacy of an International Classification of Functioning, Disability and Health (ICF)-based multidisciplinary rehabilitation concept for treatment of CRPS of the hand and to find correlations between therapy duration and outcome measures.

**Methods:**

Patients with CRPS of the hand after occupational trauma that underwent an ICF-based rehabilitation program between 2010 and 2014 were included in this retrospective study. Besides demographic data, outcomes included pain (VAS), range of motion assessed by fingertip-to-palm-distance (PTPD) and fingernail-to-table-distance (FTTD) as well as strength in grip, 3-point pinch and lateral pinch. All measures were gathered at admission to and discharge from inpatient rehabilitation therapy as well as at follow-up. Statistical analysis included paired t-test, ANOVA and Pearson's correlation analysis.

**Results:**

Eighty-nine patients with a mean age of 45 years were included in this study. Duration of rehabilitation therapy was 53 days on average. All outcomes improved significantly during rehabilitation therapy. Pain decreased from 6.4 to 2.2. PTPD of digit 2 to 5 improved from 2.5, 2.8, 2.6, and 2.3 cm to 1.3, 1.4, 1.2, and 1.1 cm, respectively. FTTD of digit 2 to 5 decreased from 1.5, 1.7, 1.5, and 1.6 cm to 0.6, 0.8, 0.7, and 0.7 cm, respectively. Strength ameliorated from 9.5, 3.7, 2.7 kg to 17.9, 5.6, 5.0 kg in grip, lateral pinch, and 3-point pinch, respectively. Improvement in range of motion significantly correlated with therapy duration. 54% of patients participated at follow-up after a mean of 7.5 months. Outcome measures at follow-up remained stable compared to discharge values without significant differences.

**Conclusion:**

The ICF-based rehabilitation concept is a reliable and durable treatment option for CRPS of the hand. Range of motion improved continuously with therapy duration and thus may serve as an indicator for optimum length of therapy.

## Background

Complex Regional Pain Syndrome (CRPS) is a rare but severe complication. Incidence varies between 5.4 and 26.4 per 100.000 person years [1, 2]. It may affect an extremity after injury, but can also involve uninjured regions of the body spontaneously. In case of CRPS of the hand, epidemiologic data show that incidence of CRPS after wrist fracture varies between 1 and 37% [3–7]. While the obligatory and leading symptom is pain, it might be accompanied by swelling, changes in skin coloration and temperature, as well as abnormal hair and nail growth [8, 9]. CRPS can sometimes be challenging to identify and it is mandatory to verify that no other cause can explain the symptoms. Disability resulting from CRPS frequently leads to an inability to work and to participate in social activities. A significant decrease in quality of life further impacts mental health and often causes depression [10]. Since it’s causation is unknown, treatment of CRPS focuses on relieving its symptoms [11, 12]. In a recent study of Miller et al., a survey among practitioners showed that more than 32 single interventions are currently utilized for treatment of CRPS [13]. Although all participants of the survey follow a multimodal approach, components of each approach vary significantly. In the majority of outpatient treatment concepts, patients suffering from CRPS receive educational interventions to facilitate self-management and interventions for pain neuroscience education. In addition, most of the multimodal therapies include physical exercises to increase range of motion and functional activity practice as well as exposure-based therapies focusing on tactile desensitization. Interestingly, multimodal therapies are often based on evidence of their single interventions only, while evidence for concepts in their entirety is lacking [14–18]. This makes it nearly impossible to estimate efficacy of current CRPS treatment concepts and predict outcomes, especially in the long-term. Furthermore, it is currently unknown how long rehabilitation therapy for CRPS patients is indicated. A good way to treat CRPS patients in a holistic biopsychological concept might be a multidisciplinary rehabilitation based on the International Classification of Functioning, Disability and Health (ICF). The ICF concept is a highly specified framework describing and organizing health condition of rehabilitation patients in a comprehensive way, providing a standard language and a conceptual basis for the definition and measurement of health and disability [19, 20]. Since its introduction in 2001 by the World Health Organization it has become the most used instrument to describe deficit of functional health condition, disability, social impairment and relevant environmental factors.

The aim of this study was to evaluate our ICF-based rehabilitation program for CRPS of the hand regarding efficacy and long-term effectiveness as well as a correlation to therapy duration.

## Methods

After obtaining institutional review board approval (Protocol no.: 837.386.17-11,220), all patients suffering from CRPS according to the Budapest criteria [21] at the upper extremity that underwent an ICF-base rehabilitation at our division of hand rehabilitation between 2010 and 2014 were included in this study. Of note, any diagnosis that could have explained CRPS symptoms, such as pain and dysaesthesia in case of a carpal tunnel syndrome, led to exclusion from this study. Detailed inclusion criteria are depicted in Table 1.

**Table 1:** Budapest clinical diagnostic criteria for CRPS [21] that served as inclusion criteria for this study

(1) Continuing pain, which is disproportionate to any inciting event
(2) Must report at least one symptom in three of the four following categories: Sensory: reports of hyperesthesia and/or allodynia Sudomotor/edema: reports of edema and/or sweating changes and/or sweating asymmetry Motor/trophic: reports of decreased range of motion and/or motor dysfunction (weakness, tremor, dystonia) and/or trophic changes (hair, nail, skin)
(3) Must display at least one sign at time of evaluation in two or more of the following categories: Sensory: evidence of hyperalgesia (to pinprick) and/or allodynia (to light touch and/or deep somatic pressure and/or joint movement) Vasomotor: evidence of temperature asymmetry and/or skin color changes and/or asymmetry Sudomotor/edema: evidence of edema and/or sweating changes and/or sweating asymmetry Motor/trophic: evidence of decreased range of motion and/or motor dysfunction (weakness, tremor, dystonia) and/or trophic changes (hair, nail, skin)
(4) There is no other diagnosis that better explains the signs and symptoms

All accidents associated with CRPS were work-related and costs for medical therapy were covered by the occupational insurance. The study was designed in accordance with the ethical standards laid down in the 1975 World Medical Association Declaration of Helsinki and its later amendments. Medical records were reviewed retrospectively regarding epidemiologic data, such as age, gender, initial trauma, time to CRPS, time-to-admission, clinical symptoms, function and duration of therapy. Clinical symptoms and function of the affected hand were evaluated at admission to and at discharge from inpatient rehabilitation as well as during outpatient follow-up visits. The latter were part of the regular clinical course after inpatient rehabilitation program and analyzed retrospectively. Functional assessment included range of motion, given in pulp-to-palm-distance (PTPD) and fingernail-to-table-distance (FTTD), both in centimeters (cm), as well as strength in grip, lateral pinch, and 3-point pinch, all three provided in kilograms (kg). Assessment of pain was further graded by the patient on a visual analog scale (VAS) from 1 to 10, with 10 being the highest possible pain felt by the participant.

### Multidisciplinary treatment approach

As soon as the diagnosis of CRPS at the hand was verified by fulfilling Budapest criteria, patients were scheduled for admission to our inpatient rehabilitation center. Time-to-admission depended on vacancy and patients’ preferences, but never exceeded three weeks. The multidisciplinary treatment concept involved physical therapists, occupational therapists, psychologists, neurologists, nuclear medicine, physicians specialized in pain therapy, and physicians specialized in hand surgery. Each patient underwent six individual sessions of physical therapy, occupational therapy, and medical training therapy as well as six group sessions of arm therapy and kinetotherapeutic bath therapy per week. Individual psychologic and pain therapy sessions were held once per week, and patients were seen by a hand surgeon twice per week. However, the therapies often have to be modified due to the actual pain intensity and ability to participate. ICF-based goal setting involved among others the goal of pain reduction, and increase in strength and ROM and was set by the patient on a weekly basis.

Physical therapy, occupational therapy, and medical training therapy focused on range of motion improvement, strength exercises, proprioception exercises, tactile and thermal desensitization techniques, mirror therapy, and massage. Psychological interventions involved mainly relaxation techniques and cognitive behavioral therapy techniques. Specialized pain therapy included pain medication according to the WHO guidelines as well as bisphosphonates, corticosteroids, anti-depressive drugs, and interventions such as brachial plexus or stellatum blockades depending on individual patient needs. ICF-based the hand surgeon coordinated and supervised the entire rehabilitation process, agreed with the patient on individual goals on a weekly basis, verified achievement of set goals and evaluated the need for surgical interventions. In addition, set goals were controlled by therapists in detailed measures of function, strength and pain.

### Statistical analysis

Patients were grouped according to therapy duration (< 3 weeks, 3—6 weeks, 6—9 weeks, > 9 weeks). Data are presented as frequencies (percentages) for the categorical variables and means—standard deviation (SD) or range for the continuous variables. Pearson correlation analysis was performed to determine the relationship between rehabilitation duration and differences in outcome measures at discharge (compared to admission) and at follow-up (compared to discharge). Pearson’s correlation coefficient was interpreted using Evans’ classification. Correlations less than 0.20 was considered very weak, 0.20 to 0.39 were considered weak, between 0.40 and 0.59 as moderate, between 0.60 and 0.79 strong, and above 0.80 as very strong. Differences in the outcome measures between admission and discharge were assessed using paired Student’s t-test (parametric). The Brown-Forsythe ANOVA test was used to test for association between rehabilitation duration and long term outcome measures (discharge to follow up visit). Data analysis was performed with GraphPad Prism version 8.3.0 for MAC (GraphPad Software San Diego, CA). Statistical significance was set at p < 0.05.

## Results

**Fig. 1:**
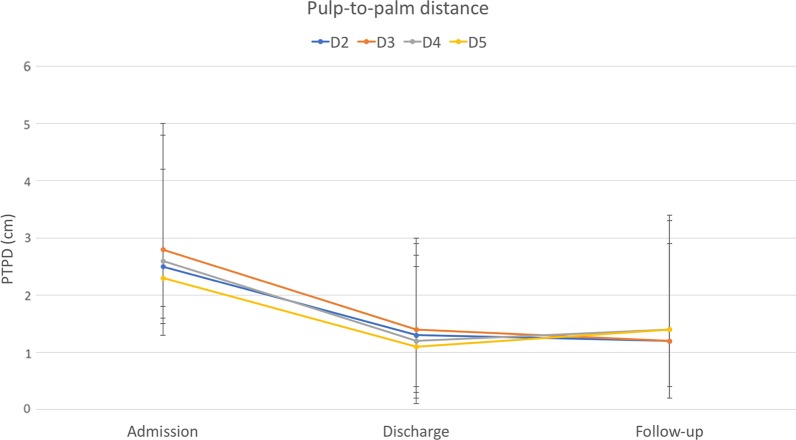
Pulp-to-palm distance of digit 2 to 5 at admission to, discharge from rehabilitation and at follow-up. Error bars depict standard deviation. X-axis: time points; Y-axis: distance in cm

**Fig. 2:**
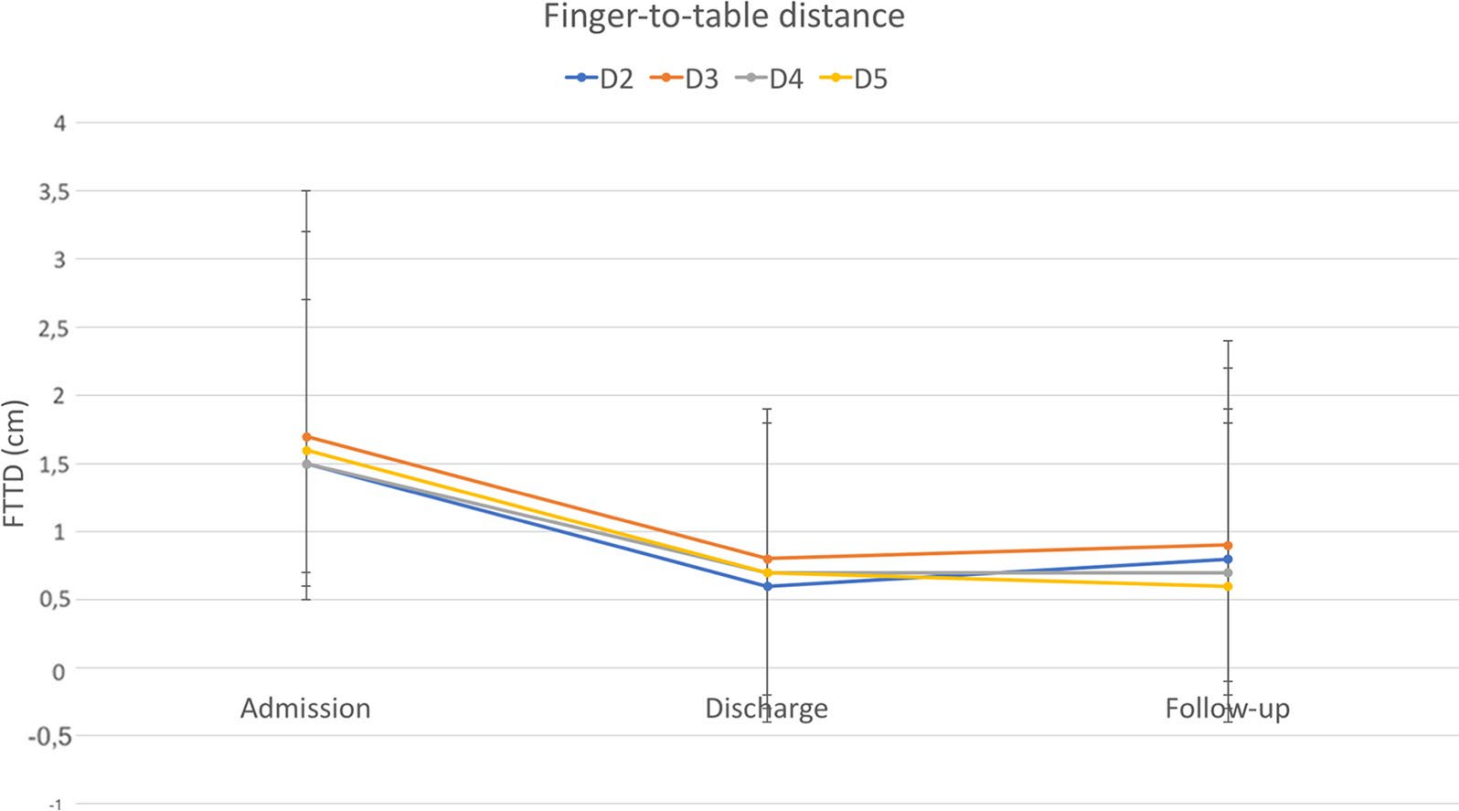
Finger-to-table distance of digit 2 to 5 at admission to, discharge from rehabilitation and at follow-up. Error bars depict standard deviation. X-axis: time points; Y-axis: distance in cm

**Fig. 3:**
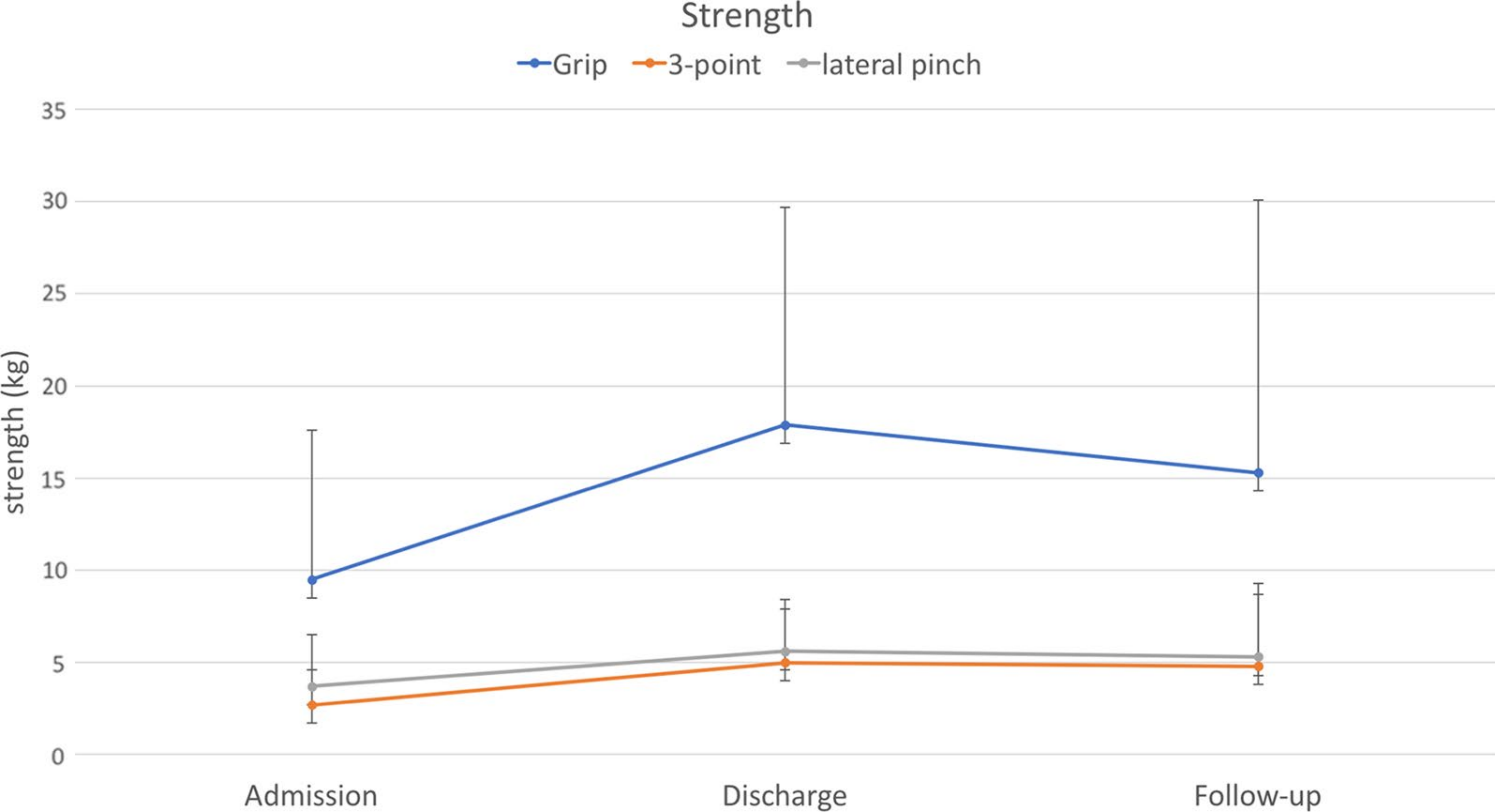
Strength in grip, 3-point pinch and lateral pinch at admission to, discharge from rehabilitation and at follow-up. Error bars depict standard deviation. X-axis: time points; Y-axis: strength in kg

**Fig. 4:**
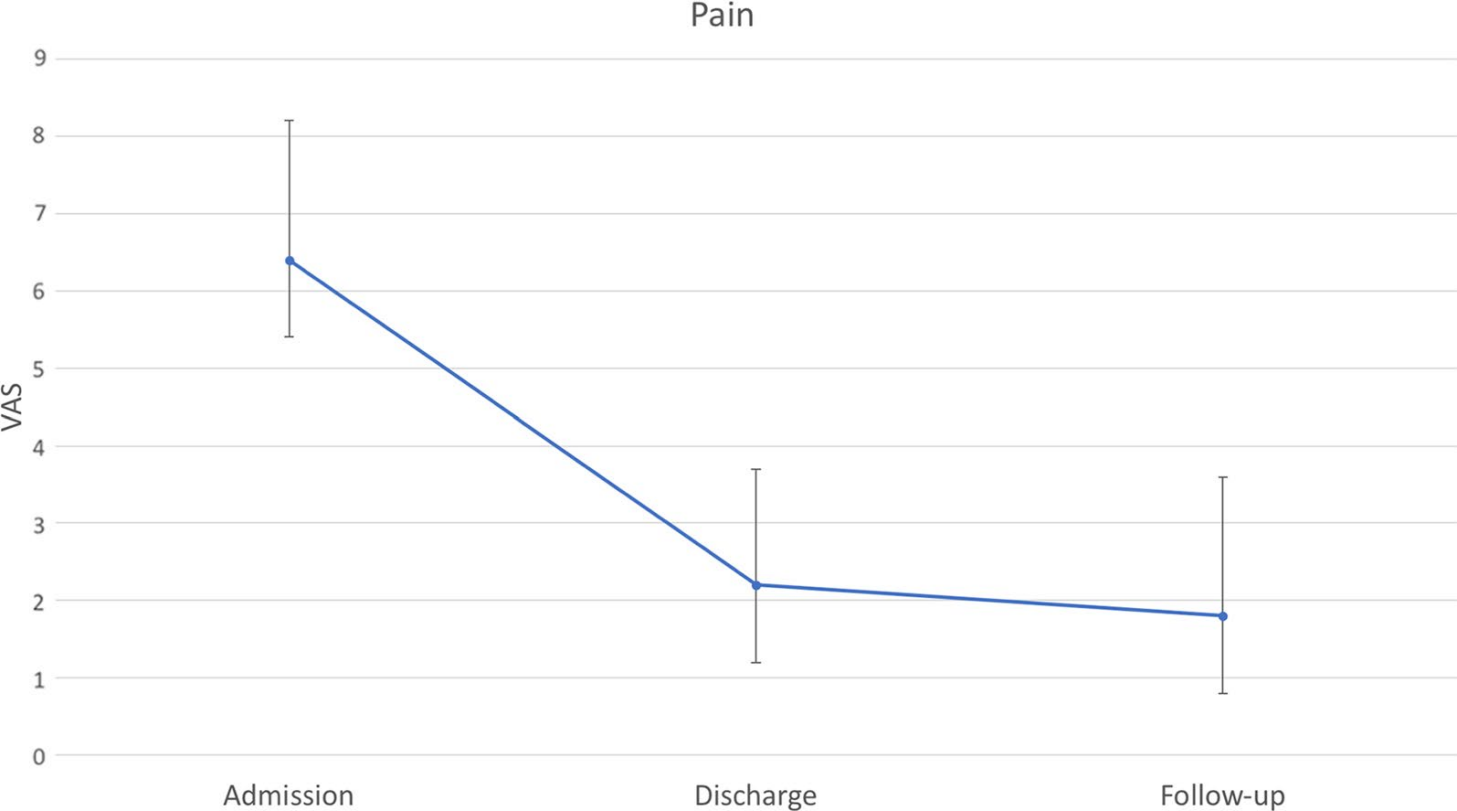
Pain on a visual analog scale from 1 to 10 at admission to, discharge from rehabilitation and at follow-up. Error bars depict standard deviation. X-axis: time points; Y-axis: Pain value form 1 to 10

From 2010 to 2014 eighty-nine patients underwent multidisciplinary ICF-based rehabilitation treatment for CRPS at the upper extremity at our facility. Fifty-five were male, and thirty-four were female. The mean age was 45.4 years, ranging from 17 to 70 years. Initial trauma was a wrist or hand fracture in 52%, tendon injury in 17%, nerve injury in 9%, sprain in 16%, and other trauma in 20% of cases (multiple diagnoses were possible). Time to CRPS diagnosis was 160 days on average, ranging from 13 to 1510 days. All patients fulfilled the Budapest criteria. Besides pain, 97% of patients suffered from swelling, 91% from hyperhidrosis, 77% from changes in skin coloration, and 81% from changes in hair growth. Epidemiologic data are given in Table 2. Six of 89 patients received plexus or Stellatum blockade as invasive procedure for pain management. Pain at admission was 6.4 on VAS (range from 2 to 10), and PTPD of digit 2 to 5 was 2.5, 2.8, 2.6, and 2.3 cm, respectively. FTTD of digit 2 to 5 was 1.5, 1.7, 1.5, and 1.6 cm, respectively. Strength at admission was 9.5, 3.7, 2.7 kg in grip, lateral pinch and 3-point pinch, respectively. The rehabilitation duration was 53.1 days on average (SD = 12.2). At discharge mean pain value was 2.2 (range 0 to 8), PTPD of digit 2 to 5 was 1.3, 1.4, 1.2, and 1.1 cm, respectively, and FTTD of digit 2 to 5 was 0.6, 0.8, 0.7, and 0.7 cm, respectively. Strength after rehabilitation was 17.9, 5.6, 5.0 kg in grip, lateral pinch and 3-point pinch, respectively. Forty-eight (54%) patients were available for follow-up. Mean time to follow-up was 7.5 months (range 1 to 27.7). Pain at follow-up was 1.8 (range from 0 to 7) and PTPD of digit 2 to 5 was 1.2, 1.4, 1.4, and 1.4 cm, respectively. FTTD of digit 2 to 5 was 0.8, 0.9, 0.7, and 0.6 cm, respectively. Strength at follow-up was 15.3, 5.3, 4.8 kg in grip, lateral pinch and 3-point pinch, respectively. Outcome measures at admission, discharge and follow-up are depicted in Table 3 and Figs. 1, 2, 3, 4. Statistical analysis of the whole study group revealed a significant increase in ROM and strength at discharge from inpatient rehabilitation therapy, whereas pain decreased significantly. In the same patient cohort, outcome measures at follow-up did not show any significant differences compared to values at discharge from interdisciplinary rehabilitation therapy (Table 3).

**Table 2:** Patient demographics

Variables	
Patients, N (%)	89 (100%)
Age, M (SD), years	45.4 ± 12.2
Gender, male, N (%)	55 (61.7%)
Initial Trauma to CRPS, M (SD), days	160.0 ± 183.0
CRPS and time to admission, M (SD), days	70.5 ± 101.5
Rehabilitation duration, M (SD), days	53.1 ± 12.2
Follow up time, M (SD), months	7.5 ± 7.0
Etiology for CRPS	
Fracture, N (%)	46 (51.7%)
Nerve injury, N (%)	8 (8.9%)
Tendon injury, N (%)	15 (16.9%)
Sprain, N (%)	14 (15.7%)
Others, N (%)	18 (20%)
Clinical evaluation	
Pain, N (%)	89 (100%)
Hyperhidrosis, N (%)	81 (91.0%)
Changes in skin coloration, N (%)	69 (77.5%)
Changes in hair growth, N (%)	72 (80.9%)

*M* mean, *SD* standard deviation, *CRPS* complex regional pain syndrome

**Table 3:** Outcome measures at admission, discharge and follow-up

Variables	Admission (A) n = 89	Discharge (D) n = 89	Follow-up visit (F) n = 48	*p-*value (A vs. D)	*p-*value (D vs. F)
PTPD Dig 2 (cm), M ± SD	2.5 ± 2.3	1.3 ± 1.6	1.2 ± 1.7	< *0.0001*	0.816
PTPD Dig 3 (cm), M ± SD	2.8 ± 2.2	1.4 ± 1.6	1.4 ± 2.0	< *0.0001*	0.747
PTPD Dig 4 (cm), M ± SD	2.6 ± 2.2	1.2 ± 1.5	1.4 ± 2.0	< *0.0001*	0.677
PTPD Dig 5 (cm), M ± SD	2.3 ± 1.9	1.1 ± 1.4	1.4 ± 1.9	< *0.0001*	0.258
FTTD Dig 2 (cm), M ± SD	1.5 ± 2.0	0.6 ± 1.3	0.8 ± 1.6	< *0.0001*	0.716
FTTD Dig 3 (cm), M ± SD	1.7 ± 2.1	0.8 ± 1.3	0.9 ± 1.7	< *0.0001*	0.789
FTTD Dig 4 (cm), M ± SD	1.5 ± 1.7	0.7 ± 1.2	0.7 ± 1.5	< *0.0001*	0.863
FTTD Dig 5 (cm), M ± SD	1.6 ± 1.6	0.7 ± 1.2	0.6 ± 1.2	< *0.0001*	0.961
Grip (kg), M ± SD	9.5 ± 8.1	17.9 ± 11.8	15.3 ± 14.8	< *0.0001*	0.511
3-point pinch (kg), M ± SD	2.7 ± 1.9	5.0 ± 2.9	4.8 ± 3.9	< *0.0001*	0.544
Lateral pinch (kg), M ± SD	3.7 ± 2.8	5.6 ± 2.8	5.3 ± 4.0	< *0.0001*	0.553
Pain (VAS) (points) M ± SD	6.4 ± 1.8	2.2 ± 1.5	1.8 ± 1.8	< *0.0001*	0.511

*PTPD* pulp to palm distance, *FTTD* fingernail to table distance, *Dig* digit, *VAS* visaul analog scale

Subgroup analysis (3 weeks, > 3–6 weeks, > 6–9 weeks, > 9 weeks) revealed statistically significant improvement of all outcome measures, irrespective of therapy duration (Table 4). Interestingly, correlation analysis showed a statistically significant association between duration of therapy and improvement in range of motion. In detail, finger motion increased continuously over the entire study period, meaning that a longer duration of therapy leads to better improvement of range of motion. In contrast, pain and strength did not show any correlation to therapy duration. In other words, pain and strength did not improve with longer inpatient therapy. Correlation analysis is depicted in Table 5. Age and gender-related differences in treatment outcome were not shown in this cohort of CRPS patients and correlation analysis of comorbidities and outcomes did not reveal any significant differences (data not shown).

**Table 4:** Influence of different rehabilitation therapy durations on fingernail-to-table distance, pulp-to-palm distance, as well as strength and pain

Rehabilitation duration	FTTD Dig 2 (cm) M ± SD	*p-*value	FTTD Dig 3 (cm) M ± SD	*p-*value	FTTD Dig 4 (cm) M ± SD	*p-*value	FTTD Dig 5 (cm) M ± SD	*p-*Value
3 weeks	− 0.88 ± 1.22	< *0.0001*	− 0.50 ± 0.63	*0.006*	− 0.73 ± 1.26	*0.029*	− 0.5 ± 0.81	*0.022*
> 3–6 weeks	− 0.71 ± 1.27	*0.02*	− 0.91 ± 1.39	*0.003*	− 1.24 ± 1.26	< *0.0001*	− 0.615 ± 1.30	*0.023*
> 6–9 weeks	− 0.8 ± 2.1	*0.009*	− 1.19 ± 1.54	*0.001*	− 1.08 ± 0.99	< *0.0001*	− 1.23 ± 1.56	*0.0005*
> 9 weeks	− 1.53 ± 2.01	*0.02*	− 1.11 ± 2.16	*0.043*	− 1.47 ± 1.92	*0.005*	− 1.11 ± 1.84	*0.020*

Rehabilitation duration	PTPD Dig 2 (cm) M ± SD	*p-*value	PTPD Dig 3 (cm) M ± SD	*p-*Value	PTPD Dig 4 (cm) M ± SD	*p-*value	PTPD Dig 5 (cm) M ± SD	*p-*value
3 weeks	− 0.79 ± 0.93	*0.003*	− 1.25 ± 1.47	*0.004*	− 1.65 ± 2.13	*0.006*	− 0.74 ± 1.26	*0.029*
> 3–6 weeks	− 0.70 ± 1.23	*0.01*	− 1.44 ± 1.28	< *0.0001*	− 1.135 ± 1.26	< *0.0001*	− 1.24 ± 1.26	< *0.0001*
> 6–9 weeks	− 1.346 ± 1.51	< *0.001*	− 1.14 ± 1.79	*0.02*	− 1.30 ± 1.31	< *0.0001*	− 1.01 ± 0.99	< *0.0001*
> 9 weeks	− 1.95 ± 1.93	< *0.001*	− 1.67 ± 2.2	*0.005*	− 1.75 ± 2.1	*0.002*	− 1.47 ± 1.92	*0.005*

Rehabilitation duration	Grip (kg) M ± SD	*p-*value	PThree-point pinch (kg) M ± SD	*p-*Value	Lateral pinch (kg) M ± SD	*p-*value	Pain (points) M ± SD	*p-*value
3 weeks	8.03 ± 7.16	*0.001*	2.02 ± 1.97	*0.0014*	1.75 ± 2.25	*0.009*	− 3.87 ± 1.69	*< 0.0001*
> 3–6 weeks	9.39 ± 9.06	*< 0.0001*	2.38 ± 1.90	*< 0.0001*	2.33 ± 2.05	< *< 0.0001*	− 4.8 ± 1.94	*< 0.0001*
> 6–9 weeks	6.70 ± 5.70	*< 0.0001*	2.31 ± 2.32	*< 0.0001*	1.39 ± 1.89	< *0.0007*	− 3.98 ± 1.87	*< 0.0001*
> 9 weeks	9.55 ± 9.32	*0.003*	2.14 ± 2.41	*0.001*	2.00 ± 3.39	*0.020*	− 3.824 ± 1.81	*< 0.0001*

*FTTD* fingernail to table distance, *Dig* digit, *M* mean, *SD* standard deviation, *PTPD* pulp-to-palm distance

**Table 5:** Correlation analysis of rehabilitation duration to outcome measures at discharge

Variables (difference A to D)	Pearson r	*p-*value
PTPD Dig 2	− 0.30	*0.005*
PTPD Dig 3	− 0.39	*0.0001*
PTPD Dig 4	− 0.40	*0.0001*
PTPD Dig 5	− 0.40	*0.0001*
FTTD Dig 2	− 0.30	*0.005*
FTTD Dig 3	− 0.24	*0.02*
FTTD Dig 4	− 0.30	*0.004*
FTTD Dig 5	− 0.25	*0.017*
Grip	0.10	0.34
3-point pinch	0.04	0.67
Lateral pinch	− 0.04	0.71
Pain	− 0.15	0.16

*A* admission, *D* discharge, *PTPD* pulp to palm distance, *FTTD* fingernail to table distance, *Dig* digit

## Discussion

In this study, we demonstrate the efficacy and long-term effectiveness of an ICF-based multimodal and interdisciplinary inpatient treatment approach for CRPS of the hand. After an intensive rehabilitation program, pain decreased significantly, and functional outcomes, such as range of motion and strength, revealed significantly better results compared to pretreatment. At long-term, follow-up pain and functional outcomes remained stable without significant differences compared to values at discharge underlining longevity of the effects gained by this multimodal and interdisciplinary inpatient rehabilitation program. Finally, correlation analysis revealed that the range of motion improved continuously with the duration of rehabilitation therapy Therefore, deficits in range of motion can serve as an indicator for extension of rehabilitation therapy.

CRPS is probably one of the most feared complications in modern hand surgery [22]. To date, no clear causality could be identified to predict its occurrence securely or to find effective ways for prevention [6]. Bean et al. searched for prognostic indicators for CRPS and determined psychological characteristics that diminished recovery capacities of patients with CRPS [23]. In a mixed-effects model, the authors found a correlation between anxiety and pain-related-fear with poorer outcomes of CRPS treatment. Patients with these characteristics might be more affected by CRPS and thus should undergo treatment earlier. Buller et al. found a marginal increase in the incidence of CRPS when carpal tunnel release was performed in the same procedure with fasciotomy for Dupuytren’s contracture [24]. This was contradicting previous reports that highly recommended separating both surgeries due to significantly higher CRPS rates. The authors concluded that carpal tunnel release and fasciotomy for Dupuytren’s contracture could be combined if necessary. Searching for further hand related pathologies associated with CRPS, our own group found a statistically significant co-prevalence of CRPS and carpal tunnel syndrome (CTS) in a cohort of 791 patients. Interestingly, if CTS was treated adequately, rehabilitative therapy was significantly shorter compared to patients suffering from CRPS of the hand without coinciding carpal tunnel syndrome [25].

In this study, all patients had a history of hand trauma, of which fracture of the wrist or hand showed the highest incidence. Although this could indicate a higher correlation of CRPS with hand fractures compared to other hand trauma, however, to verify this hypothesis, the relative frequency of hand fractures would be necessary and thus cannot be proven in this study. In contrast, outcome measures as indicator for treatment efficacy were correlated to the type of initial hand trauma. However, no statistically significant correlation could be identified in this subgroup analysis (data not shown). Furthermore, finding baseline pathologies or co-prevalence of hand related trauma was not the scope of this study, rather evaluating long-term outcomes and its’ correlation to therapy duration of an ICF-based rehabilitation program. The ICF was used as a framework to help structure rehabilitation plans and for defining treatment goals.

Recently published guidelines of the United States, United Kingdom and the Netherlands recommend a multimodal treatment to overcome CRPS [14–16]. While physical therapy can be found in all recommendations, only the Dutch society declares patient education as mandatory. In a recent survey of Miller et al. 132 therapists provided insights into nowadays clinical practice for CRPS treatment [13]. In contrast to the aforementioned guidelines, patient education was part of the treatment strategy in more than 80% of cases. Furthermore, the majority of participants stated that physical exercise interventions, especially to improve range of motion and strength are part of their CRPS treatment concept. With respect to effectiveness of these interventions Cochrane systematic review provided evidence that both physiotherapy and occupational therapy have positive effects in CRPS patients [26]. In detail, graded motor imagery and mirror therapy are effective in decreasing pain and functional disability, whereas multimodal physiotherapy improves impairment in general [27]. Of note, 30% of CRPS patients are non-responders to CRPS treatment. Although we could not detect any true non-responders in this patient cohort, outcomes would significantly ameliorate, if the worst 30% of patients would be subtracted from the study population. This interesting aspect further underlines our findings and substantiates the efficacy of the shown inpatient CRPS treatment approach.

Educational training for self-management and pain neuroscience is a pillar of our rehabilitation concept. Both physical therapists and psychologists work with the patient in individual sessions and additional group sessions on effective ways to decrease pain and increase function. Furthermore, physical exercise interventions for range of motion and strength improvement are an essential component of our rehabilitation strategy. In our opinion, treatment of CRPS is only effective in a multidisciplinary and multimodal approach.

Interestingly, none of the current guidelines provides recommendations regarding the length of CRPS therapy. Common practice is to commence the therapy as early as possible and to continue as long as improvement is evident. Elomaa et al. introduced an integrated interdisciplinary therapy of 12 weeks duration and showed that some symptoms of CRPS improved, such as pain and function, however, distress and quality of life remained unchanged [28]. The authors concluded that the multimodal therapy approach is effective and recommendable but the duration is too short to achieve better results. Of note, the multimodal therapy approach was in an outpatient setting and details about quality and frequency of single interventions were not provided.

In our study, we found out that outcome measures pain, strength and range of motion significantly improved already after 3 weeks of inpatient multidisciplinary intensive rehabilitation therapy. While pain and strength did not show further improvement with therapy duration, range of motion ameliorated gradually with therapy duration. Therefore, range of motion can be used as a marker for therapy duration by indicating capacities or limitations for improvement. It remains unclear, if an inpatient therapy is necessary to achieve superior results in CRPS treatment. This question should be addressed in the future. However, many patients were referred from all over Germany and thus distances from home too far for an outpatient setting. In addition, total therapy duration exceeded seven hours on some days, making travels challenging for patients that are already handicapped by CRPS.

One limitation of this study is the retrospective approach. However, data were gathered independently from multiple therapists involved in the patients’ rehabilitation program. Furthermore, participation at follow-up was rather low with 54% of patients. Nevertheless, efficacy of the ICF-based multidisciplinary inpatient rehabilitation program was validated in 89 patients and with means of several in-depth outcome measures, thus good quality data is sufficient to draw a conclusion. Another aspect that needs to be considered is the effect of workers’ compensation on long term outcome measures [29–36]. However, at least in our study population this effect was not evident, underlining the long-term effectiveness of our ICF-based multimodal and multidisciplinary therapy approach.

Finally, late diagnosis of CRPS and associated delay in treatment can lead to worse outcomes. Therefore, current practice is to make an early diagnosis and start therapy as soon as possible. In this study, some patients with delay in diagnosis were initially presented to other clinics or private practices, so that it was not possible to investigate reasons for the delay in diagnosis and foremost differentiate the delay in diagnosis from a timely adequate diagnosis with a late on set of CRPS. Furthermore, we compared late diagnosis in CRPS from early diagnosis in this study, but could not find any significant differences in outcome measures (data not shown). We believe that this is due to the fact that some patients with a late diagnosis suffered from a late onset of CRPS and thus received a timely adequate diagnosis and treatment.

## Conclusion

In this study we demonstrated that an ICF-based multidisciplinary inpatient rehabilitation program is effective in improving symptoms of CRPS at the hand. Pain, function and strength ameliorated significantly and outcomes remained stable during long-term follow-up. In addition, range of motion significantly correlated with therapy duration, and thus can serve as good indicator for therapy cessation or extension, respectively.

## Data Availability

All data is included in the manuscript.

## Abbreviations

CRPS: Complex regional pain syndrome
ICF: International Classification of Functioning, Disability and Health
VAS: Visual analog scale
ROM: Range of motion
PTPD: Fingertip-to-palm-distance
FTTD: Fingernail-to-table-distance
ANOVA: Analysis of variance
SD: Standard deviation
Cm: Centimeters
Kg: Kilograms
CTS: Carpal tunnel syndrome
